# Fatal arrythmia in a young man after COVID-19 vaccination: An autopsy report

**DOI:** 10.1097/MD.0000000000037196

**Published:** 2023-02-02

**Authors:** Hiroshi Minato, Akane Yoshikawa, Sho Tsuyama, Kazuyoshi Katayanagi, Satoaki Hachiya, Keisuke Ohta, Yasuhiro Myojo

**Affiliations:** a Department of Diagnostic Pathology, Ishikawa Prefectural Central Hospital, Kanazawa City, Ishikawa Prefecture, Japan; b Department of Emergency and Critical Care, Ishikawa Prefectural Central Hospital, Kanazawa City, Ishikawa Prefecture, Japan.

**Keywords:** case report, COVID-19, fatal arrythmia, myocarditis, vaccination

## Abstract

**Rationale:**

The benefits of COVID-19 mRNA vaccination are claimed to be substantial; however, vaccination-related myocarditis and pericarditis have also been observed globally, particularly among young men. In most cases, the symptoms are mild and resolve on their own; however, fatal cases have rarely been described.

**Patient concerns:**

A healthy 40-year-old Japanese man suddenly experienced tachycardia and lost consciousness 2 days after vaccination. Continued resuscitation recovered the spontaneous heartbeat; however, the patient did not regain consciousness and died 9 days later. Electrocardiography after resuscitation showed marked ST-segment depression and incomplete right bundle branch block. Influenza antigen and polymerase chain reaction tests for SARS-CoV-2 were negative.

**Diagnoses:**

Fatal arrhythmia after a second COVID-19 mRNA vaccination.

**Interventions:**

We performed an autopsy and studied the material morphologically and immunohistochemically.

**Outcomes:**

At autopsy, several small inflammatory foci with cardiomyocytic necrosis were scattered in the right and left ventricles, with a propensity for the right side. Some inflammatory foci were located near the atrioventricular nodes and His bundles. The infiltrating cells predominantly consisted of CD68-positive histiocytes, with a small number of CD8-positive and CD4-positive T cells. In this case, myocarditis was focal and mild, as is mostly observed following COVID-19 mRNA vaccination. However, the inflammatory foci were close to the conduction system and were considered the cause of fatal arrhythmia.

**Lessons:**

Although the benefits of COVID-19 vaccination appear to outweigh the side effects, it should be noted that fatal arrhythmias may rarely occur, and caution should be taken if individuals, particularly young men, complain of any symptoms after vaccination.

## 1. Introduction

The benefits of COVID-19 mRNA vaccination are claimed to be substantial, especially for those over the age of 12 years, and the benefits exceed the risks.^[1]^ However, vaccination-related myocarditis and pericarditis have also been observed globally, particularly among young men.^[1]^ In most cases, the symptoms are mild and resolve on their own; however, fatal cases have rarely been described.^[2–4]^ Here, we present an autopsy case of a patient with myocardial inflammation who had a fatal arrhythmia 2 days after receiving the second COVID-19 mRNA vaccination. To the best of our knowledge, no report has focused on the association between histological localization of cardiac inflammatory foci and deadly arrhythmias in COVID-19 vaccine-associated myocarditis.

Informed consent was obtained from the patient’s wife for the publication of this case report and accompanying images in accordance with local ethical approval requirements and in accordance with the Declaration of Helsinki.

## 2. Case report

### 2.1. Clinical course

**Figure 1.**
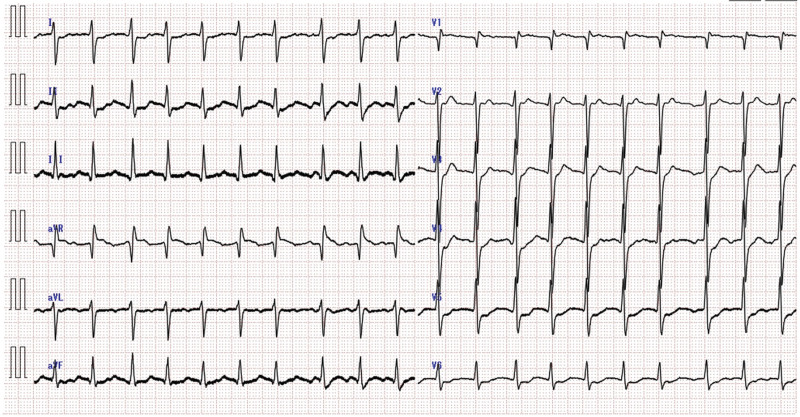
Electrocardiogram after heartbeat resumed. Marked ST depression and incomplete right bundle branch block were observed.

A Japanese man in his early 40s developed a fever the day after his second vaccination with the COVID-19 vaccine (Pfizer-BioNTech COVID-19 mRNA vaccine). He suddenly lost consciousness the next day and rushed to the emergency room. He had a history of atopic dermatitis in childhood but was otherwise healthy. Electrocardiography revealed ventricular tachycardia that transitioned to pulseless electrical activity with defibrillation. Continuous resuscitation resumed spontaneous heartbeat; however, the patient did not regain consciousness. Despite mechanical ventilation, percutaneous cardiopulmonary support, and temperature control, multiple organ failure progressed due to irreversible brain damage caused by hypoxic encephalopathy. The patient died on the 9th day of hospitalization. On his recovered heartbeat, the electrocardiography showed marked ST-segment depression and an incomplete right bundle branch block (Fig. 1). His body mass index was 32.7 and his temperature was 37.1 °C. Laboratory data showed increased white blood cell count 11.9 × 10^3^/µL (reference range, 3.3–8.6), troponin T 1.9 ng/mL (0–0.029), C-reactive protein 1.38 mg/dL (0–0.14), creatinine kinase 692 U/L (59–248), CK-MB 38 ng/mL (0–5), lactate dehydrogenase 1143 U/L (124–222), and brain natriuretic peptide 59.8 pg/mL (0–18.4). Eosinophilic count 1.5% (0–8.5), red blood cells 5.55 × 10^6^/µL (4.35–5.55), hemoglobin 16.1 g/dL (13.7–16.8) and platelet count 213 × 10^3^/µL (158–348) were within normal ranges. The patient was influenza antigen-negative and had negative polymerase chain reaction test results for SARS-CoV-2.

### 2.2. Pathological findings

**Figure 2.**
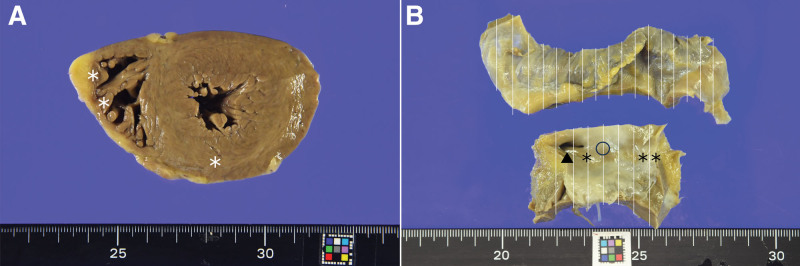
The heart was hypertrophic, weighed 495 g, and there were no signs of myocardial infarction. (A) Small inflammatory foci were scattered in both the right and left ventricle with a propensity for right side. (B) Inflammatory foci (*, **) were observed near the atrioventricular node (〇) and His bundle. ▲: coronary ostium.

**Figure 3.**
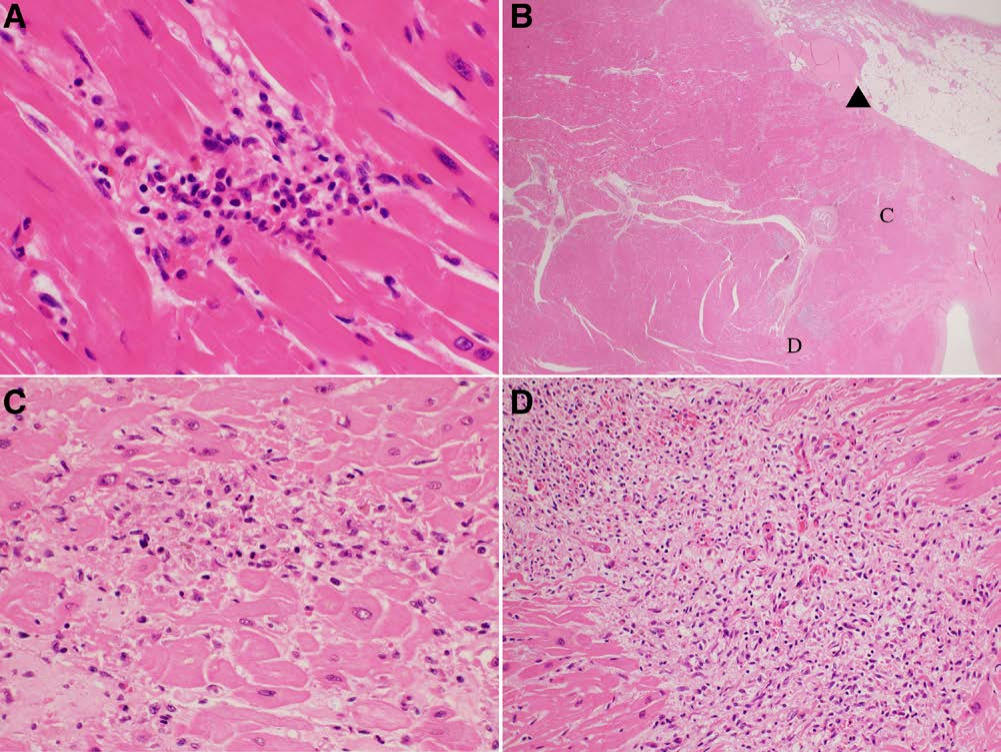
Histology of myocardial inflammation. (A) Focal lymphocytic and some eosinophilic infiltration is seen in the myocardium with myocytic degeneration. (B) Several inflammatory foci were found near the atrioventricular (A–V) node (▲). (C) An inflammatory focus near the (A–V) node shows myocardial necrosis and infiltration of lymphocytes. (D) Some inflammatory foci showed granulation changes with an increase in capillaries and infiltration of lymphocytes and histiocytes.

**Figure 4.**
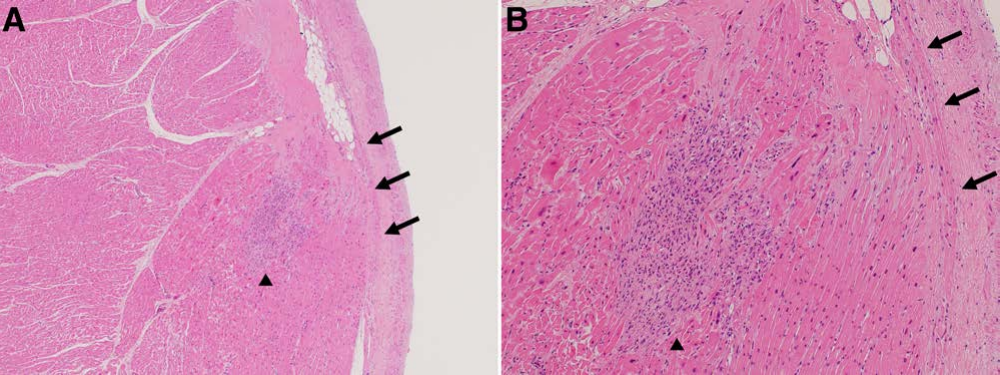
(A) Inflammatory focus (▲) vicinity of the His bundle (→). (B) The focus was composed of granulation tissue with lymphohistiocytic infiltration.

**Figure 5.**
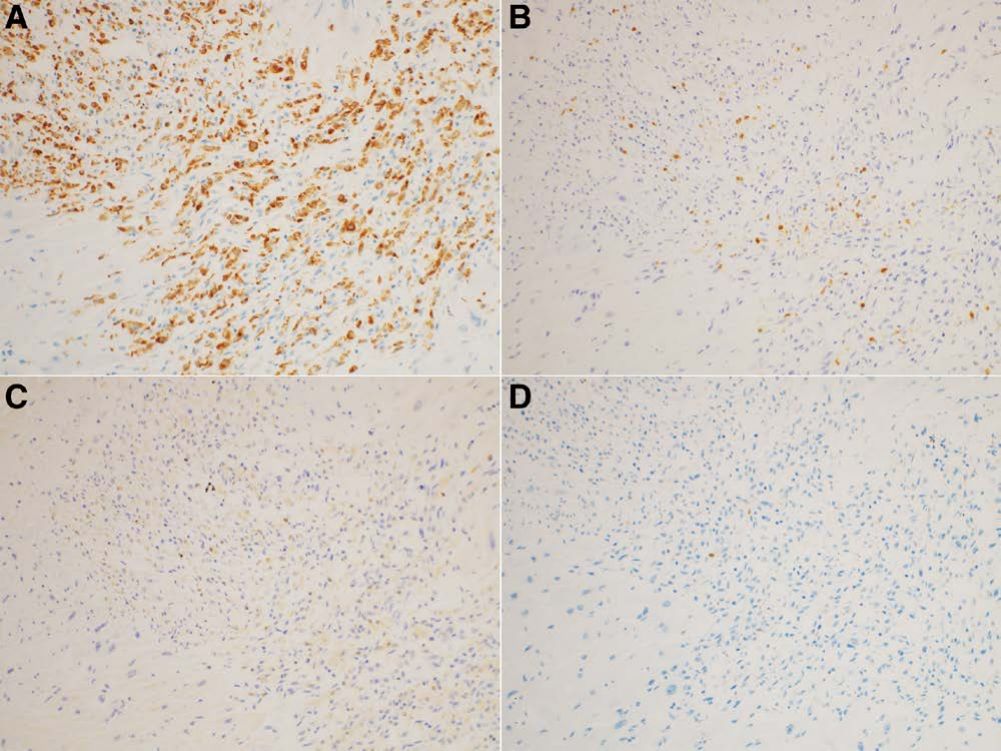
Inflammatory infiltrates were predominantly composed of CD68-positive histiocytes (A), mixed with a smaller number of CD8-positive T cells (B). CD4-positive T-cells (C) and CD20-positive B-cells (D) were relatively few, and CD138-positive plasma cells were not observed.

At autopsy, the heart weighed 495 g, without myocardial infarction (Fig. 2). No significant coronary artery stenosis was observed. Heart histology revealed several small inflammatory foci scattered in both the right and left ventricles, with a propensity toward the right side (Fig. 2A). Some inflammatory foci were located near the atrioventricular node and His bundles (Figs. 2B and 3). Inflammatory foci showed myocardial degeneration, necrosis, or granulation changes, with an increase in small blood vessels (Fig. 3). Some inflammatory foci contained eosinophils (Fig. 3A). No multinucleated giant cells or granulomatous changes were observed. Although the number of inflammatory foci was relatively small, their location suggested that they may have caused lethal arrhythmia (Figs. 3 and 4). Cardiomyocytes did not show marked hypertrophy or disarray and no features were suggestive of cardiomyopathy. Inflammatory infiltrates were predominantly composed of CD68-positive histiocytes mixed with a small number of CD8-positive T cells. There were relatively few CD4-positive T cells and CD20-positive B cells, and no CD138-positive plasma cells were observed (Fig. 5). The left axillary lymph node was swollen to 32 mm, but the normal structure of the lymph node was preserved, suggesting reactive changes associated with the vaccination. Minute latent papillary carcinoma measuring 3 mm and chronic lymphocytic thyroiditis were observed in the thyroid gland. The lungs were congested and edematous, and congestion was observed in the spleen, liver, and kidneys. Moderate steatosis was observed in the liver tissues. Multiple organ failure due to hypoxic encephalopathy is thought to be a cause of death.

## 3. Discussion

The frequency of myocarditis and pericarditis after COVID-19 mRNA vaccination was much higher than after non-COVID-19 vaccination. According to the Vaccine Adverse Event Reporting System database, the most common vaccination associated with myocarditis is COVID-19, which accounts for 87% of the top 10 vaccinations related to postvaccination myocarditis, followed by smallpox (12%), anthrax (3.5%), and others (0–1% range).^[5]^

According to Vaccine Adverse Event Reporting System statistics, as of June 2021, the frequency of myocarditis/pericarditis within 7 days after the second dose of the COVID-19 mRNA vaccine was higher than that in the general population of men aged 12 to 49 years and women aged 12 to 29 years. In particular, 18 to 24-year-olds was the most common age group for both men and women, with men at 50.5/1 million (expected value max 1.84) and women at 4.39/1 million (expected value max 1.15).^[1]^ The incidence peaked 3 days after the second vaccination, with most cases occurring on days 2 to 4.^[6]^ According to a report by the Ministry of Health, Labor and Welfare in Japan, as of November 4, 2021, in the vaccinated Japanese population aged 10 to 29 years, myocarditis-related events were reported in 159 men and 15 women. In contrast, in the general population, only 21 men and 13 women per 1 million aged 30 to 49 years and 15 men and 12 women aged ≥ 50 years were reported to have myocarditis-related events.^[7]^ a trend that is common among young men worldwide.

However, the reason why myocarditis is more common in men remains unclear. Various factors such as hormonal balance and environmental factors are conceivable. Estrogen may play a protective role, whereas testosterone may promote myocarditis.^[8]^ A similar mechanism may be involved in vaccine-induced myocarditis.

Viral infection and vaccines promote the production of T-bet, a Th1 transcription factor, from T follicular helper cells, which regulate immune responses by differentiating CD8+ T cells from effector T cells into memory T cells. T-bet induces and suppresses inflammation, and an imbalance in T-bet function causes autoimmune disease. An experiment in mice reported that loss of T-bet promoted IL-17 production and caused autoimmune myocarditis.^[9]^ In humans, the T-bet levels have been shown to increase with age.^[10]^ Mormile speculates that, in addition to low T-bet expression in young people, T-bet polymorphisms may generate autoreactive CD8 cells that increase the likelihood of myocarditis.^[11]^ Estrogen may upregulate T-bet expression, making myocarditis less likely.^[12]^

Myocarditis following COVID-19 mRNA vaccination is usually mild and has good prognosis. However, occasional deaths have occurred.^[2,6,13–15]^ In Israel, 95% of patients have mild illness, but 0.7% die.^[6]^ The myocarditis in our case was histologically categorized as mild; however, its proximity to the conduction system was considered to have caused fatal arrhythmia.

Few studies have reported the histological findings of post-COVID-19 vaccination myocarditis. Previous reports have mainly described the infiltration of CD68-positive histocytes and lymphocytes^[4]^; however, the infiltration of eosinophils, neutrophils, and multinucleated giant cells has also been identified.^[2,4,16]^ In this case, histiocytes predominated, and CD8-positive T-cells outnumbered CD4-positive cells. Several studies have described a similar histology.^[17–19]^ In contrast, other studies have claimed that CD4 is more prominent than CD8.^[2,20]^ The infiltration of T cells and histiocytes appears to be the key component of inflammation; however, whether CD4 or CD8 is prevalent may depend on the chronology of inflammation. Many reports have indicated that inflammation is focal, which was also true in this case.^[14,20]^ In some reports of patients with clinically suspected myocarditis, myocardial biopsies did not reveal inflammation.^[21,22]^ This may be because the inflammatory foci are scattered in the myocardium and cannot be biopsied. Interestingly, some reports state that inflammation is centered in the right ventricle and septum, which is consistent with our case.^[14,20]^ Some studies argue that this phenomenon is similar to catecholamine-induced damage and toxic cardiomyopathy,^[23]^ but the patient did not exhibit confluent areas of necrosis. Myocarditis following COVID-19 mRNA vaccination may be clinically or histologically heterogeneous.

The pathogenesis of postvaccination myocarditis remains unclear.^[3,15]^ One theory is that the immune system is activated by the recognition of mRNA as an antigen.^[24]^ The other theory is that antibodies against the spike glycoprotein contained in the vaccine cross-react with α-myosin and transglutaminase to elicit an immune response.^[17,25]^ In this case, chronic thyroiditis and fatty liver were observed, and the possibility that this background contributed to the immunological abnormalities against the vaccine cannot be completely ruled out.

In this case, a direct causal relationship between COVID-19 mRNA vaccination and myocarditis could not be definitively established because we did not test for viral genomes or autoantibodies in the tissue specimens. However, no other cause has been identified to indicate the etiology of myocarditis. COVID-19 mRNA vaccine-associated myocarditis was strongly implicated in this case based on the patient’s sex, age, time of onset, and histology.

In conclusion, we reported a case of lethal arrhythmia with myocarditis that developed after COVID-19 mRNA vaccination. Although rare, myocarditis may occur after vaccination, and caution should be exercised if people complain of symptoms, such as fever, cough, chest pain, dyspnea, or syncope after vaccination, especially in young men. Although the severity of myocarditis associated with COVID-19 mRNA vaccines is usually mild and the benefits of vaccination appear to outweigh the side effects, it should be noted that fatal arrhythmia may occur depending on the site of inflammatory foci in the heart.

## Author contributions

**Conceptualization:** Hiroshi Minato.

**Data curation:** Hiroshi Minato, Akane Yoshikawa, Satoaki Hachiya.

**Formal analysis:** Sho Tsuyama, Keisuke Ohta.

**Investigation:** Hiroshi Minato, Akane Yoshikawa, Satoaki Hachiya.

**Project administration:** Hiroshi Minato, Yasuhiro Myojo.

**Resources:** Akane Yoshikawa, Sho Tsuyama, Satoaki Hachiya, Keisuke Ohta.

**Supervision:** Kazuyoshi Katayanagi, Keisuke Ohta, Yasuhiro Myojo.

**Validation:** Sho Tsuyama, Kazuyoshi Katayanagi, Keisuke Ohta, Yasuhiro Myojo.

**Visualization:** Hiroshi Minato, Akane Yoshikawa, Satoaki Hachiya.

**Writing – original draft:** Hiroshi Minato.

**Writing – review & editing:** Hiroshi Minato, Satoaki Hachiya.
